# Active inference and the anatomy of oculomotion

**DOI:** 10.1016/j.neuropsychologia.2018.01.041

**Published:** 2018-03

**Authors:** Thomas Parr, Karl J. Friston

**Affiliations:** Wellcome Trust Centre for Neuroimaging, Institute of Neurology, University College London, 12 Queen Square, London WC1N 3BG, UK

**Keywords:** Free energy, Saccades, Oculomotor, Brainstem, Predictive coding, Active inference

## Abstract

Given that eye movement control can be framed as an inferential process, how are the requisite forces generated to produce anticipated or desired fixation? Starting from a generative model based on simple Newtonian equations of motion, we derive a variational solution to this problem and illustrate the plausibility of its implementation in the oculomotor brainstem. We show, through simulation, that the Bayesian filtering equations that implement ‘planning as inference’ can generate both saccadic and smooth pursuit eye movements. Crucially, the associated message passing maps well onto the known connectivity and neuroanatomy of the brainstem – and the changes in these messages over time are strikingly similar to single unit recordings of neurons in the corresponding nuclei. Furthermore, we show that simulated lesions to axonal pathways reproduce eye movement patterns of neurological patients with damage to these tracts.

## Highlights

• Eye movement control is cast as an inferential process that induces Newtonian rotational forces.
• A plausible computational anatomy of Bayesian filtering in the brainstem is proposed.
• Smooth pursuit and saccadic eye movements are simulated.
• Lesions to the model cause similar patterns of eye movements to those found in neurological patients.

## 1 Introduction

There are many neurological (Sereno and Holzman, 1995, Büttner et al., 1999, Perry and Zeki, 2000, Anderson and MacAskill, 2013) and psychiatric (Holzman and Levy, 1977, Lipton et al., 1983, Sereno and Holzman, 1995) conditions that cause impairments of eye movement control. As such, assessment of oculomotion forms a crucial part of any neurological examination. We aim to characterise the functional anatomy of eye movement control by appealing to active inference, a principled approach to describing Bayes optimal behaviour (Friston et al., 2009). Our agenda here is to try and understand the oculomotor system in terms of its computational anatomy, as a complement to similar attempts to understand the control of eye movements at higher levels of the visual system; e.g., (Itti and Koch, 2001, Bruce and Tsotsos, 2009).

Previous active inference accounts of eye movements have focused on saccadic target selection (Mirza et al., 2016, Friston et al., 2017b) and ignored the mechanics of oculomotion, or have made use of the simplifying assumption that the position of the eyes can be altered directly through simple attractor dynamics (Friston et al., 2012, Friston et al., 2017a). Here, we follow the example of models that have treated the eyes as physical objects, subject to Newton's laws (Robinson, 1964, Robinson, 1968, McSpadden, 1998, Adams et al., 2012, Perrinet et al., 2014). We build upon these models by equipping each eye with separate kinetics, which are predicted by the brain using a model that is common to both eyes. We emphasise the anatomy and electrophysiology that emerge from this theoretical treatment and their striking resemblance to the properties of the brainstem (Büttner-Ennever and Büttner, 1988, Büttner and Büttner-Ennever, 2006).

The oculomotor system is a crucial interface between inferential processes of the brain, and the Newtonian world that it inhabits. It forms a distributed network (Parr and Friston, 2017a) that involves the cerebral cortex (Paus, 1996, Corbetta et al., 1998), the cerebellum (Berretta et al., 1993), and the basal ganglia (Hikosaka and Wurtz, 1985b, Hikosaka et al., 2000). Ultimately, neuronal messages from these regions combine to generate signals to the extraocular muscles to move the eyes. It is the brainstem that performs the translation of these instructions into motor nerve signals (Sparks, 1986, Sparks, 2002, Sparks and Mays, 1990). In this paper, we seek to understand the computations that must be performed to do this, and their neurobiological substrates. We begin by describing the mechanics of the eyes. We then provide an overview of the principles of active inference, and use these to motivate a predictive (generative) model of eye movements. We demonstrate through simulation that this reproduces eye movements consistent with health and disease, and show the emergence of established electrophysiological observations from these simulations.

## 2 Mechanics of eye movements

**Fig. 1:**
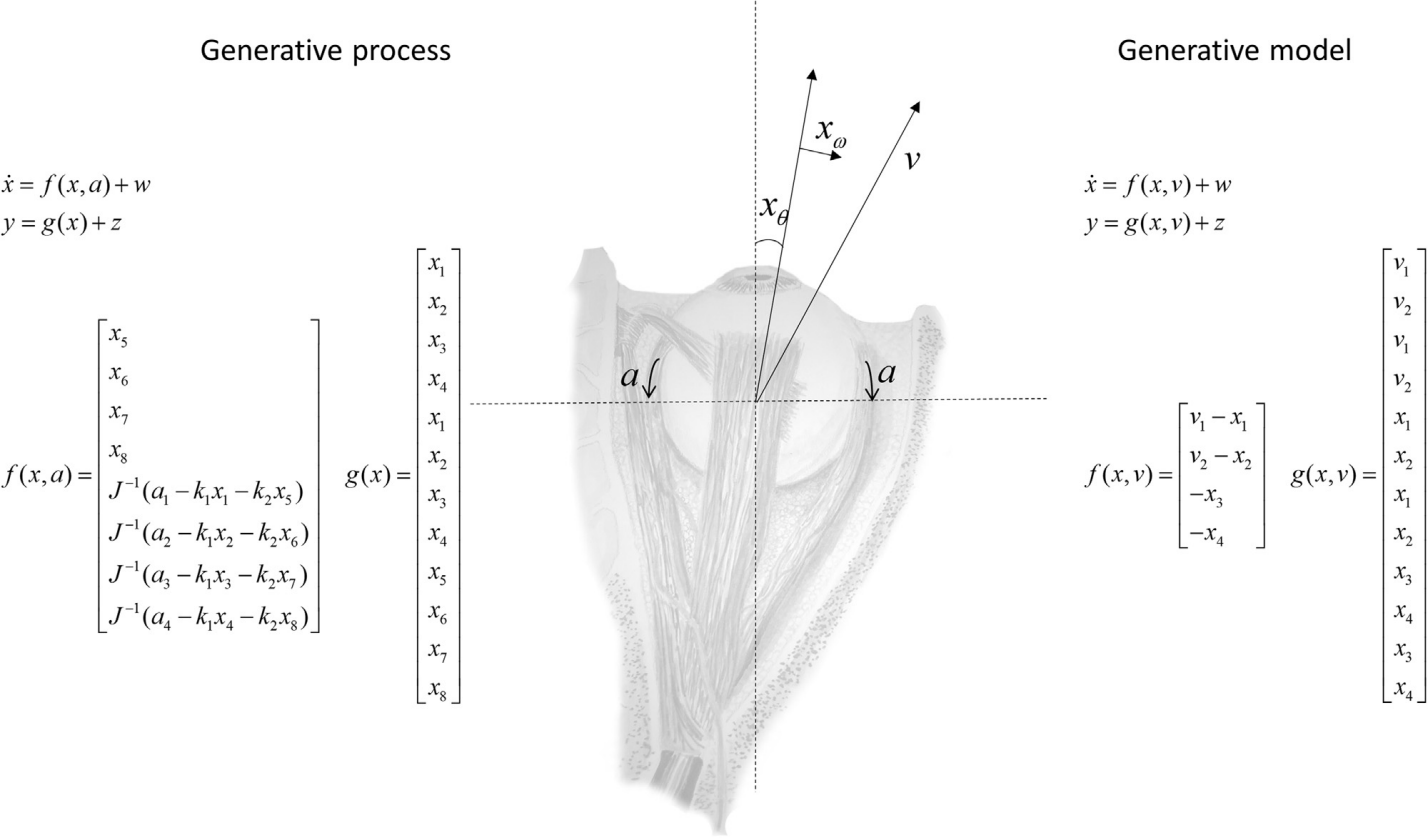
**Equations of motion** This schematic shows the equations used to determine the motion of the eyes, and the sensations they generate. On the left, the pair of equations defining the ‘real-world’ generative process are shown. On the right, the analogous equations are shown for a generative model of that process. Note that the dimension of the sensory data, y, is equal for both, but the dimensions of the hidden states, x, differ. In the generative process, $x_{1,2,3,4}$ are the (2 × 2) angular horizontal and vertical positions for the right and left eye (components of the $x_{\theta }$ vectors). $x_{5,6,7,8}$ are the angular velocities (components of the $x_{\omega }$ vectors). Each of these is associated with a result torque involving the extraocular muscles, $a_{1,2,3,4}$, an elastic torque with spring constant $k_{1}$, and a viscous torque with a viscosity constant $k_{2}$. The resultant torque is converted to acceleration through division by the moment of inertia of the eyeballs J. In the generative model, $x_{1,2}$ are the horizontal and vertical positions of both eyes, which are crucially assumed to be the same. $x_{3,4}$ are the velocities. $v_{1,2}$ are the two components of the target fixation vector. w and z are random Gaussian fluctuations with means of zero and precisions of $\Pi_{x}$ and $\Pi_{y}$ respectively.

Saccadic eye movements implement the transition from one stationary fixation to another. While we may select a new target for fixation, the physical world does not allow us to alter position directly. Instead, changes in position must be brought about by applying forces that accelerate the eyes towards their target. We will first discuss the influence of these forces, and consider the translation of a desired location into forces in the next section. For simplicity, we assume only two forces acting on each eye. These are resultant forces in the horizontal and vertical dimensions. Each force gives rise to a torque, made up of an active term (muscle contraction), an elastic term, and a viscous term. Using Newton's second law in its rotational form, we arrive at the equations of motion shown on the left of Fig. 1. These equations are relatively simple, but could in principle be replaced by a set of more realistic equations that take account of, among other things, the non-linear relationship between muscle elasticity and length (McSpadden, 1998).

In addition to the equation describing the movement of the eyes themselves, it is necessary to specify how the angular position and velocity of each eye gives rise to sensory data. The information carried from the eye to the brainstem can be classified into two broad categories. Visual information is passed through the optic nerve (Cranial nerve II), while proprioceptive data from the extraocular muscles travels through afferent fibres in the oculomotor nerves (CN III, IV, VI). We have assumed a simple visual signal in this paper: it is generated through an identity mapping, with added noise, from the position of the eyes (Faisal et al., 2008). In other words, what the eyes see depends entirely on where they look.

The nature of proprioceptive signals from the extraocular muscles is a controversial topic (Donaldson, 2000), but the presence of muscle spindles – the sensory organs of proprioception – in human extraocular muscles has been convincingly demonstrated (Cooper and Daniel, 1949), as has the type of reflex associated with these spindles in other muscles (Sherrington, 1893). It is worth acknowledging that the structure of these spindles is simpler than those found in other muscles (Ruskell, 1989), but the density is comparable (Lukas et al., 1994). In most skeletal muscle, afferent nerve fibres from the muscle spindles carry data about the velocity (type Ia afferents) and instantaneous length of a muscle (type II afferents). Similar signals have been recorded from the oculomotor nerve (Cooper et al., 1951, Tomlinson and Schwarz, 1977), when the extraocular muscles are stretched. We therefore assume that there are two proprioceptive modalities from each eye, carrying signals analogous to the II (position) and Ia (velocity) afferent fibres. Each of these has a horizontal and a vertical component. The equations determining these outputs are shown on the left of Fig. 1. Having specified these primary afferents, we turn to the treatment of these sensory signals by the brain.

## 3 Active inference

The Free energy principle states that living systems must minimise their variational free energy over time (Friston et al., 2006, Friston, 2009). The Free energy is an upper bound on surprise – or negative log evidence – so this is equivalent to the (almost tautological) statement that organisms are ‘self-evidencing’ (Hohwy, 2016), and seek out the sensory data that maximises the evidence for their own existence. For example, humans exist only within narrow range of temperatures. Sensing a temperature that is comfortably within this range carries greater evidence for existence than one outside it, so the free energy principle mandates that humans should act to ensure the former (Bruineberg et al., 2016). Minimisation of free energy through action and perception is referred to as active inference. The equivalence between active inference and self-evidencing can be seen through Jensen's inequality (Beal, 2003):

F(y~,q)⏟FreeEnergy=−Eqlnp(y~,x~,v~)q(x~,v~)≥−lnEqp(y~,x~,v~)q(x~,v~)⏟Jensen'sinequality=−lnp(y~)⏟Negativelogevidence:q=argminF

In the above, p is a probability distribution that defines the beliefs an organism has about the way in which sensory data is generated. q is an arbitrary probability distribution that approximates a posterior probability distribution when the free energy is minimised. We refer to v as hidden causes, while x are latent or hidden states. The sensory data y is the only set of variables an organism has access to. The tilde notation implies generalised coordinates of motion (Friston et al., 2008), $\tilde{y}=vec(y,y\prime ,y\prime \prime ,\ldots )$, a vector of temporal derivatives. This defines the trajectory of a variable in the same way as a Taylor series. With these definitions, we can write the equations of active inference as gradient descents on the variational free energy.

$$\begin{array}{c} {\dot{\tilde{\mu }}}_{v}=D{\tilde{\mu }}_{v}-\partial_{{\tilde{\mu }}_{v}}F\\ {\dot{\tilde{\mu }}}_{x}=D{\tilde{\mu }}_{x}-\partial_{{\tilde{\mu }}_{x}}F\\ \dot{a}=-\partial_{a}F \end{array}$$

The notation $\partial_{u}≜\frac{\partial }{\partial u}$ is used to simplify the equations above. $\mu_{v}$ and $\mu_{x}$ are the means (expectations) of the approximate posterior distributions of v and x, respectively. D is a block diagonal matrix with components

$$D_{ij}=\left\{ \begin{array}{cc} 1 & \mathrm{if}\;\;j-i=1\\ 0 & \mathrm{otherwise} \end{array}\right..$$

The foregoing provides a brief account of a very general formulation of (self evidencing) systems that effectively infer the causes of their sensory input to suppress surprise – or maximise Bayesian model evidence. Technically, the first pair of equations above corresponds to a generalised (Bayesian) filter. In this setting, a ‘filter’ is a process that recovers latent or hidden states from observed signals. However, the last equation changes the game profoundly. This is because it describes action on the generative process – that changes the ‘filtered’ signals, as we will see below. It is clear from this formulation that we must compute the free energy gradients in the above equation to perform a gradient descent. To do this, we have to define the joint distribution $p(\tilde{y},\tilde{x},\tilde{v})$ that expresses an organism's beliefs about the processes that generate its sensations – its generative model.

## 4 Generative model

To specify the generative model, we factorise the joint distribution above to give

$$\begin{array}{c} p(\tilde{y},\tilde{x},\tilde{v})=p(\tilde{y}|\tilde{x},\tilde{v})p(\tilde{x}|\tilde{v})p(\tilde{v})\\ p(\tilde{y}|\tilde{x},\tilde{v})=N\left(\tilde{g},{\tilde{\Pi }}_{y}\right)\\ p(\tilde{x}|\tilde{v})=N\left(\tilde{f},{\tilde{\Pi }}_{x}\right)\\ p(\tilde{v})=N\left(\tilde{\eta },{\tilde{\Pi }}_{v}\right) \end{array}$$

This factorisation rests on a pair of equations, f and g, analogous to those in the generative process above: one that determines the temporal dynamics of the system, and one that determines how the system gives rise to sensory data. These are depicted on the right of Fig. 1. $\tilde{\eta }$ is the mean of the prior distribution over $\tilde{v}$, and ${\tilde{\Pi }}_{v}$ is its precision (i.e., inverse covariance matrix).

**Fig. 2:**
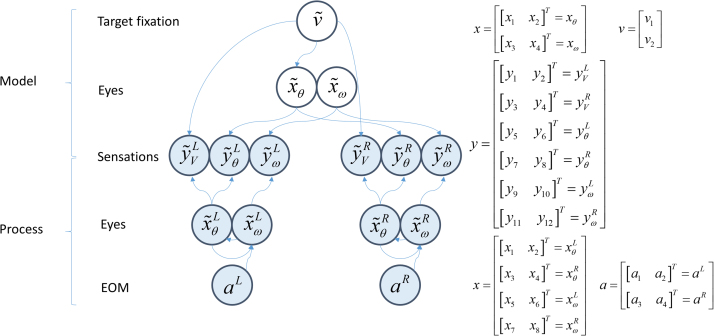
**The interface between model and process** This Bayesian network shows how the generative process (filled circles) gives rise to sensory data, and how the generative model (unfilled circles) proposes this data is generated. Arrows connecting two variables indicate that the second variable is conditionally dependent on the first. Note that, as described in the main text, action of the extraocular muscles (EOM) in the real world causes changes in velocity (i.e. accelerations); while fictive fixation locations cause changes in position in the generative model. The relationship between the vectors in this graph and the variables of Fig. 1 are shown on the right.

The interface between the generative model and process is illustrated in the Bayesian network in Fig. 2, and this highlights the important differences between the two. The model is much simpler than the process. This is because the model does not allow for each eye to move independently, whereas the position of one eye offers no constraint over that of the other in the physical world. The other key differences are that action is part of the generative process, while hidden causes are only found in the model. The former causes changes in angular velocity, while the latter changes angular position. The hidden cause acts as a point attractor, drawing the eyes towards this position.

**Fig. 3:**
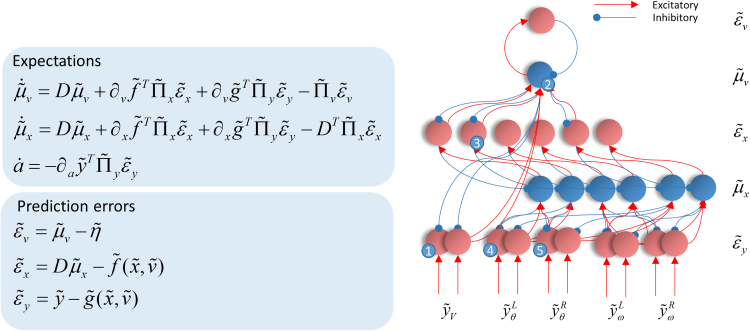
**Neuronal message passing** On the left are the equations describing a gradient descent on variational free energy. On the right, we show how these equations map to a neuronal message passing scheme for the generative model outlined above. To do so, we have simply assigned the terms on the left hand side of each equation to a neuronal population, and mapped the influences between each population with excitatory and inhibitory connections. We have separated the states representing positions and velocities into right and left components; for consistency with the representation of each hemifield on the contralateral side of the sagittal plane in the brain. The numbers in little blue circles refer to the anatomical designation of expectation and error units in Fig. 5.

If we compute the free energy gradients using a generative model of the form outlined above (Appendix), they can be substituted into the gradient descent equations to arrive at the differential equations in Fig. 3 (Friston et al., 2010). On the right hand side of Fig. 3, we illustrate how these equations could be implemented by passing messages between populations of neurons (Friston and Kiebel, 2009, Bastos et al., 2012, Shipp, 2016). Ascending messages here are (excitatory) prediction errors, while descending messages are (inhibitory) predictions. It is this pattern that characterises predictive coding (Rao and Ballard, 1999, Friston and Kiebel, 2009).

**Fig. 4:**
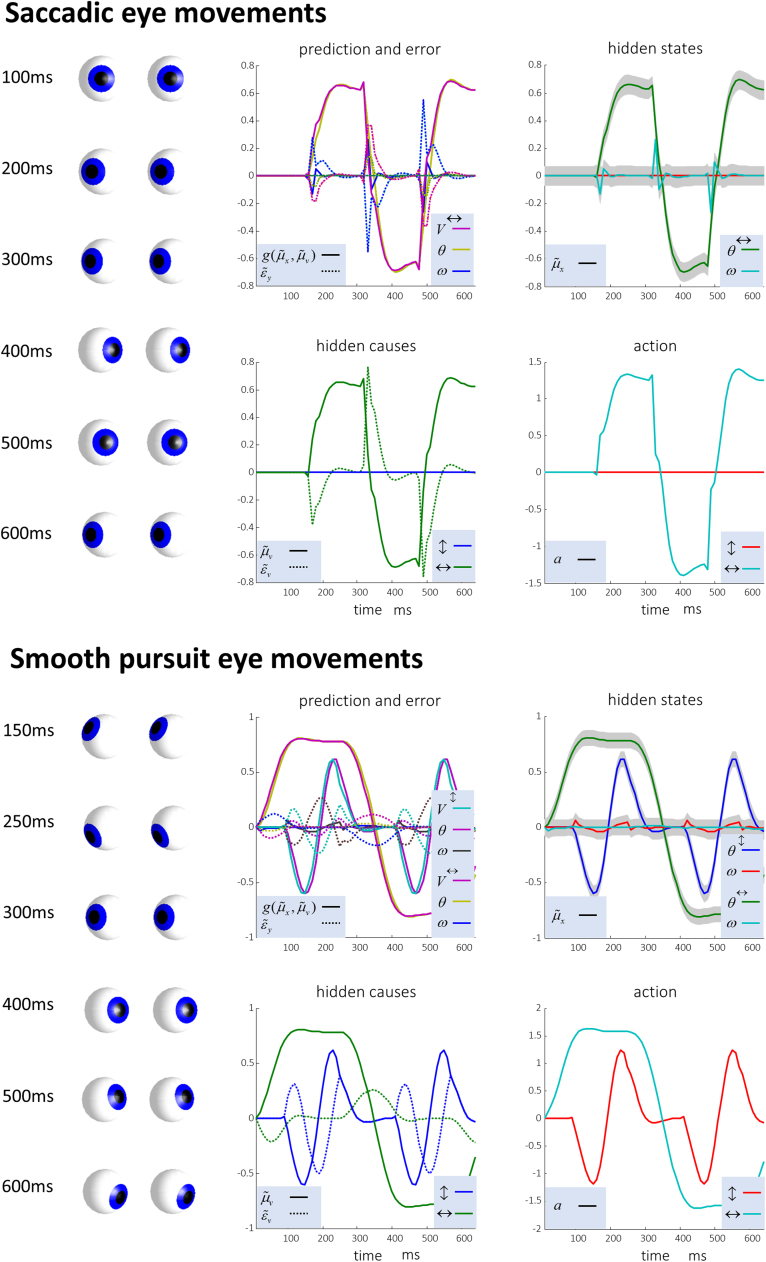
**Simulated eye movements** These plots show the changes in expectations (solid lines) and prediction errors (dotted lines) over time for the hidden causes and states during saccadic eye movements (upper), and smooth pursuit movements (lower). The eye positions at various times are shown on the left of each set of plots. The grey regions correspond to 90% Bayesian confidence intervals around the inferred hidden states; namely the vertical and horizontal angular positions and velocities. The legend in the lower right of each plot indicates the modality represented by each line (visual = V, type II afferent/position = θ, type Ia afferent/velocity = ω). For example, a dotted line with a colour associated with V represents a prediction error in the visual domain. To see the key variables plotted individually, please refer to Fig. 5, where these are represented in separate raster plots.

Fig. 4 shows the results of applying these equations, with two different prior distributions over the trajectory of a fictive fixation location. The first is a discontinuous function that changes discretely to different values, inducing saccades. The second is a sinusoidal function that gives rise to smooth pursuit eye movements. For both priors, the active inference scheme successfully computes the forces required to fulfil these beliefs. The common generative model for both eyes ensures the eye movements are conjugate – i.e. the eyes move together. In summary, using a plausible generative model and standard (active inference or filtering) dynamics we can reproduce the control of eye movements. Notice that we have not appealed to any control theory: in active inference, motor control follows naturally from the suppression of prediction errors generated by prior expectations: see Fig. 3. In other words, the active filter has prior beliefs about where it should be looking and action fulfils those beliefs in a Bayes optimal fashion. The plausibility of this sort of scheme has been addressed in the context of visual search (Friston et al., 2012) and oculomotor delays (Perrinet et al., 2014).

We now turn to the question of the biological substrates of the active filtering equations used to generate oculomotor behaviour per se.

## 5 Anatomy and electrophysiology

**Fig. 5:**
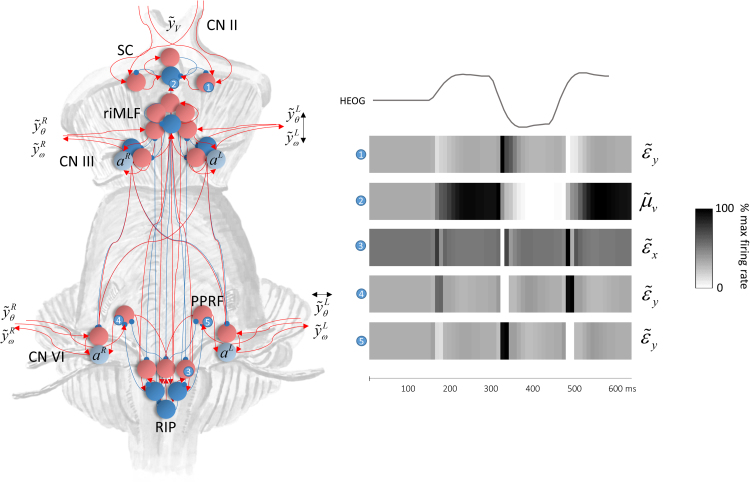
**The computational anatomy of oculomotion** On the left of this schematic, we show a plausible anatomical implementation of the Bayesian filtering equations in Fig. 3. This satisfies the connectivity constraints described in the main text. Note that we have included motor neurons (grey) that represent action. As Fig. 3 indicates, these only receive direct influences from the prediction error units at the sensory level. On the right, we show the simulated neuronal activities, along with a horizontal electrooculographic (HEOG) trace indicating the eye position. Each of the numbered raster plots is associated with a particular neuronal population indicated by numbers in little blue circles. See the main text for a description of these units and Fig. 3 for their equivalent location in the computational architecture. SC = superior colliculus; riMLF = rostral interstitial nucleus of the medial longitudinal fasciculus; PPRF = parapontine reticular formation; RIP = raphe interpositus nucleus.

The biological implementation of the equations in Fig. 3 is anatomically constrained in several ways. First, sensory inputs must reach the brain by the cranial nerves that carry that information. The neuronal populations that receive these inputs directly must reside in regions of the brain that contain the terminals of the relevant sensory afferent fibres. Similarly, neurons encoding actions should be lower motor neurons that contribute efferent fibres to the cranial nerves. The abducens nucleus mediates movements in the horizontal dimension only, and all movements in the vertical dimension are mediated by cranial nerves originating in the midbrain. The computational anatomy shown in Fig. 5 satisfies these constraints, and is remarkably consistent with the patterns of excitatory and inhibitory connectivity of the brainstem (Parr and Friston, 2017b).

To illustrate the neuronal plausibility of this computational anatomy, electrophysiological responses of cells in each region were simulated by taking the representations of each variable, as shown in the plots in Fig. 4, and converting them into raster plots. The first two raster plots in Fig. 5 show the firing rates of two of the three neuronal populations in the superior colliculus. The colliculus contains cells with three distinct electrophysiological phenotypes: ‘burst’, ‘fixation’, and ‘build-up’ cells (Munoz and Wurtz, 1995a). Burst cells fire at the start of a saccade, as can be seen in the first raster plot. This cell type is known to disnaptically inhibit cells in the Raphe nucleus interpositus (RIP) (Yoshida et al., 2001). This is consistent with the computational anatomy here, as there is an excitatory connection to a second collicular population that has inhibitory connections to the RIP. Both physiologically and anatomically, this cell type appears to be consistent with prediction error units signalling visual prediction (or ‘retinal-slip’) errors of the type implicated in models of eye movement (Krauzlis and Lisberger, 1989).

Fixation cells are active while a fixation is maintained. The second firing rate plot shows a cell that is active maximally only during fixations in one direction. These cells are known to project directly to cells in the RIP (Gandhi and Keller, 1997), again showing consistency with our proposed anatomy. These cells appear to signal the expected hidden cause. Build-up cells have yet another distinct phenotype, and must be assigned to the only remaining collicular cell type in Fig. 5, which signals the error in the expected hidden cause. We discuss this cell type in more detail below, but first turn to a key target of projections from the superior colliculus.

The RIP contains a population of cells known as ‘omnipause’ cells (Büttner-Ennever et al., 1988). These cease firing at the start of a saccade, but are active during fixations. This corresponds well to the third raster plot that shows a decrease in activity locked to each saccade. This signal is the prediction error related to the hidden states encoding current eye position. Neurons in the RIP inhibit those in the rostral interstitial nucleus of the medial longitudinal fasciculus (thought to coordinate vertical saccades (Büttner-Ennever and Büttner, 1978)) and in the parapontine reticular formation (that coordinates horizontal saccades (Cohen et al., 1968, Henn, 1992)) (Strassman et al., 1986). The fourth and fifth rows of raster plots show neurons in the latter area. These neurons show bursting activity that triggers a saccade, here related to the error in positional (proprioceptive) sensations. We have simulated such neurons representing saccades to either side of space.

**Fig. 6:**
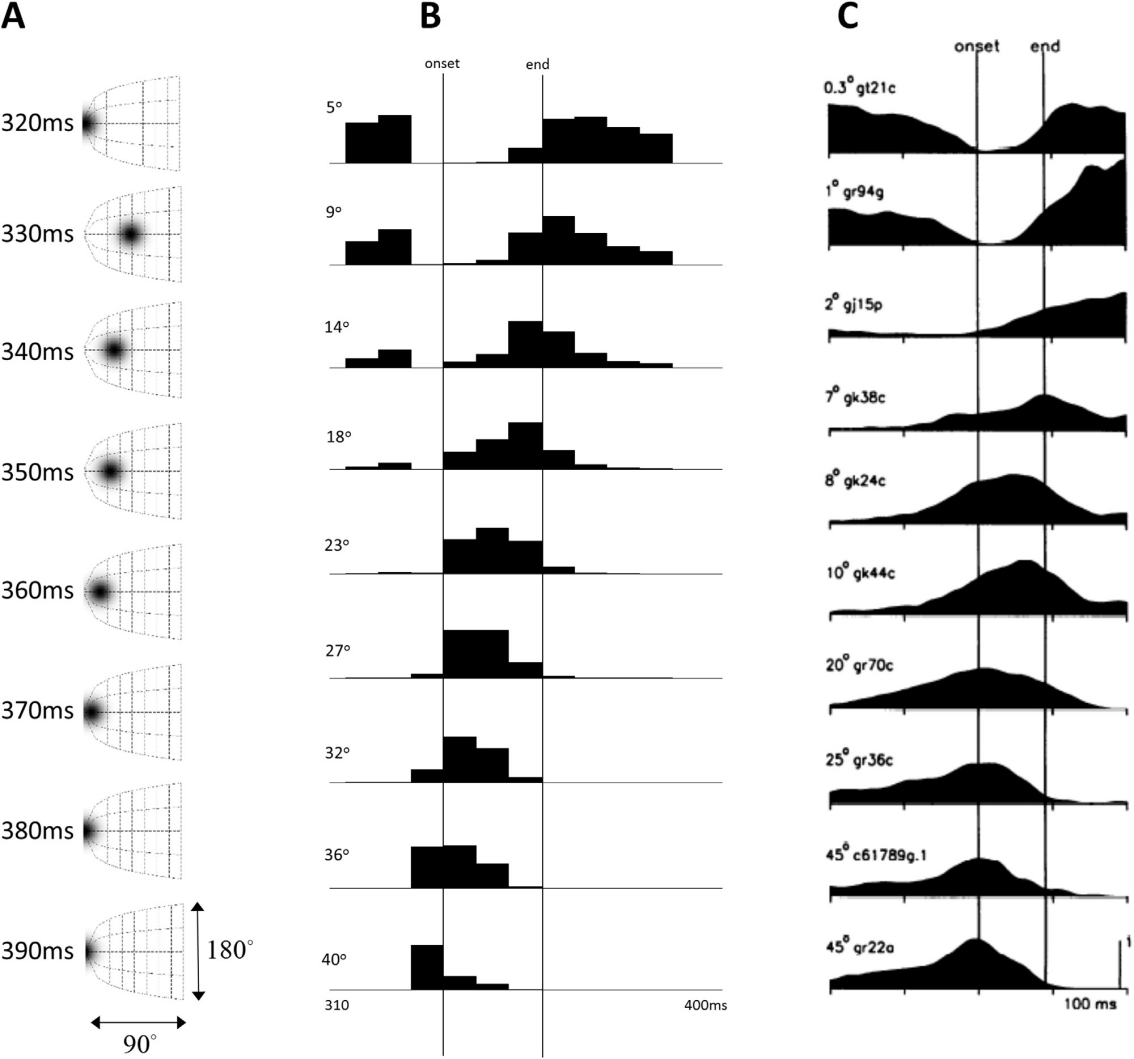
**Collicular ‘build-up’ cells** This shows the population activity in collicular build-up cells during one of the saccades illustrated in Fig. 4 (left). Our simulated build-up cells are those that signal the error in the hidden cause (target fixation location). A shows this as if we had imaged the right superior colliculus, which represents the left side of space. We have made use of the known retinotopy of the colliculus (Quaia et al., 1998) to plot this activity. B shows a set of simulated recordings of single cells from the onset to end of the saccade. Each cell represents a different retinotopic location, indicated by the angles given for each plot. Note that the eccentricity increases with each row. C shows real data (adapted from Munoz and Wurtz, 1995b) from single unit recordings of build-up cells in the superior colliculus.

The pattern of activity of the build-up cells is very interesting, when viewed at a population level (Lee et al., 1988, Munoz and Wurtz, 1995b). To simulate the spatiotemporal characteristics of electrophysiological responses in collicular build-up cells during saccades, we treated the retinotopic location vectors (i.e. the horizontal and vertical components of the error) as encoding the peaks of activity in the superior colliculus. This enabled us to generate simulated responses of colliculus neurons in which (Gaussian) ‘bumps’ of activity moved over a retinotopic map, similar to those elicited in computational models of the superior colliculus (Bozis and Moschovakis, 1998, Seung, 1998, Seung et al., 2000, Trappenberg et al., 2001, Richert et al., 2013). In turn, this enabled us to simulate spatiotemporal responses that would have been observed (by assuming a fixed shape of bump); either by imaging perisaccadic population responses in the deep layers of the superior colliculus (see Fig. 6A) – or unit responses at any particular location – over time – in terms of perisaccadic time histograms (see Fig. 6B). The post stimulus (saccade) time histograms bear a remarkable similarity to empirical results of the sort shown in Fig. 6C (Munoz and Wurtz, 1995b).

## 6 Lesions

**Fig. 7:**
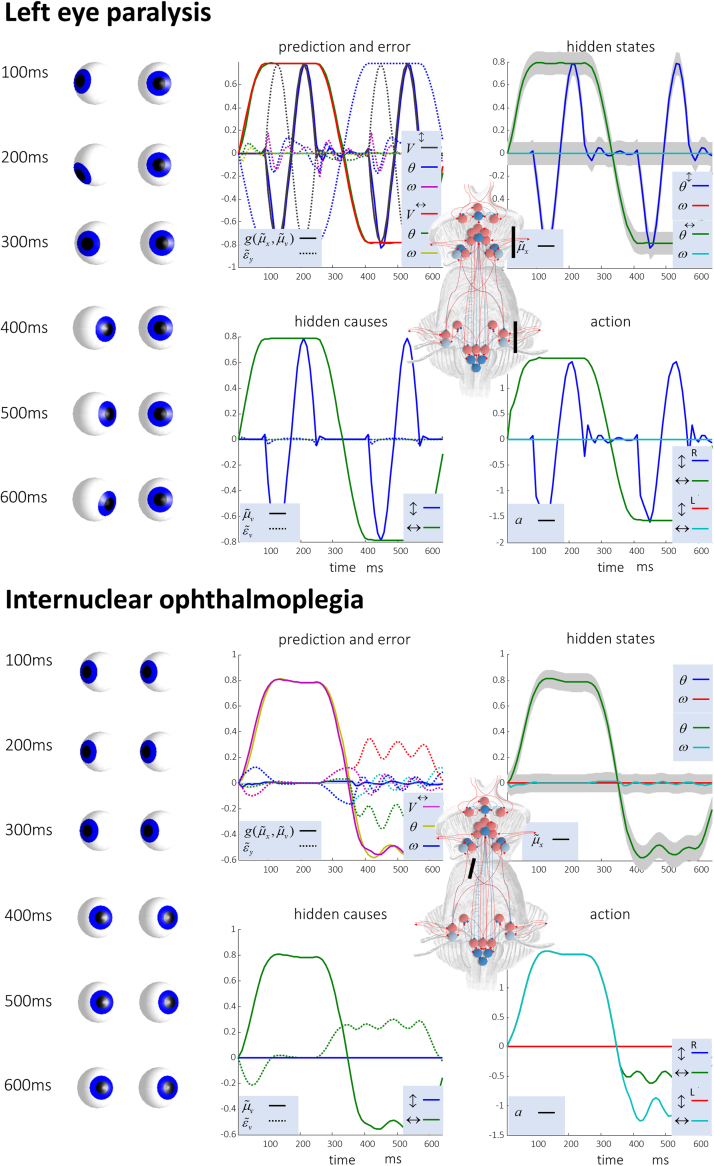
**Computational lesions** These plots demonstrate the consequences of simulated lesions. The first is a lesion of all the connections between the brainstem and the extraocular muscles of the left eye. As both the plots and the simulated eyes show, this causes a paralysis of the left eye, in keeping with what we would expect. On the right, we show the consequences of a lesion to the medial longitudinal fasciculus. The images and the plot of ‘action’ show that rightward gaze occurs normally in both eyes, but that leftward gaze reveals a deficit. The right eye fails to adduct to the same degree as the left abducts, and this induces nystagmus in both eyes – primarily the left. This is known clinically as an internuclear ophthalmoplegia. Please see refer to Fig. 4 for an explanation of these plots.

Having demonstrated the anatomical and physiological plausibility of an active inference formulation of oculomotor control, we used the anatomical constraints underwriting the computational anatomy in Fig. 5 to motivate simulated lesions. Our first lesion removed all the connections that travel in the oculomotor cranial nerves on the left. This is to demonstrate that the simulation reproduces sensible results; i.e. the paralysis of the left eye (Fig. 7, left). Computationally, this disconnection precludes the receipt of sensory data by proprioceptive prediction error units for the left eye, and disconnects action units from the extraocular muscles.

The second simulation aims to model a subtler lesion: damage to the medial longitudinal fasciculus, that travels from the abducens (CN VI) nucleus in the pons to the contralateral oculomotor (CN III) nucleus in the midbrain, causes a clinical sign referred to as an ‘internuclear ophthalmoplegia’. This is commonly seen in demyelinating conditions, such as multiple sclerosis, that induce white matter lesions. This pathology represents a disconnection syndrome (Catani and ffytche, 2005) that manifests as a failure of conjugate control of eye movements.

Fig. 7 (right) shows the results of performing this lesion *in silico*. Our lesion disrupts the signal from the left CN VI to the right CN III (see Fig. 5). Computationally, this represents a disconnection between error and expectation units encoding horizontal positional error and angular velocity respectively. As in real patients, both eyes are able to look to the right normally. However, when looking to the left, the left eye is able to look laterally, but the right eye fails to keep up while moving medially. This violation of conjugacy induces nystagmus in the (healthy) left eye. In our simulation, nystagmus is seen in both eyes, but more the left than the right. The deficit is most obvious in the plot labelled ‘action’.

## 7 Discussion

We have demonstrated in the above that, given a prior belief about anticipated fixation locations, $\tilde{\eta }$, Bayesian filtering can be used to generate movements that fulfil these beliefs. An important outstanding issue relates to the source of these priors. In predictive coding, there are typically higher hierarchical levels in play that send descending messages (predictions) to the lower level (Kiebel et al., 2008). These are used to derive the (empirical) prior beliefs at the lower level. In short, in this paper we have focused on the lowest level of deep (hierarchical) active vision that translates predictions about "where I am going to look next" into oculomotion that realises these predictions. As the predictions $\tilde{\eta }$ enter the Bayesian filtering equations to form prediction errors ${\tilde{\varepsilon }}_{v}$, any descending connections would have to target units encoding these prediction errors. The anatomy of connections to the superior colliculus therefore hints at the anatomy of higher levels generating top-down predictions (Parr and Friston, 2017a). This anatomy includes projections from the frontal eye fields (Fries, 1984), the parietal cortex, and the substantia nigra pars reticulata (Hikosaka and Wurtz, 1983). We will attempt to address the role of these connections in future work, and to link them to the decision processes we have previously attributed to cortical and subcortical regions (Parr and Friston, 2017a). This will be essential in order to account for more complex, oculomotor behaviour, including the spatial patterns of saccadic searches their resemblance to ‘Lèvy flights’ (Brockmann and Geisel, 1999, Roberts et al., 2013).

We note that there are some subtle differences in the neuronal responses we have simulated (Fig. 5) compared to those measured in real neurons. For example, our simulated burst neurons show not only an increase in firing before a saccade in a given direction, but also a decrease in firing rate before a contralateral saccade. When these neurons have been interrogated in vivo (Munoz and Wurtz, 1995a), a directional sensitivity of this type has been demonstrated. The firing rate of a burst neuron is higher when a saccade is performed in one direction compared to a saccade in the opposite direction. However, there is no clear decrease in activity, relative to baseline firing rate, in response to a saccade contralateral to the preferred direction of a burst neuron – as seen in our simulations. There are several possible explanations for this discrepancy. One is that, as firing rates cannot be negative, the positive and negative parts of the variables encoded by our synthetic neurons are actually represented by different groups of burst neurons. A second possibility is that the mapping between these variables and neuronal firing rates is a convex function. If this is the case, we would expect very small changes in firing rate for a change in a variable at the lower end of the scale compared to those induced by the same change at higher values. The low baseline firing rate of burst neurons (Munoz and Wurtz, 1995a) supports this interpretation.

In addition to the oculomotor syndromes simulated here, an interesting next step would be to consider a broader range of pathologies. For example, schizophrenia is a psychiatric disorder associated with subtle oculomotor abnormalities, including changes in smooth pursuit eye movements (Thaker et al., 1998). Previous research using this form of modelling has been useful in characterising this kind of deficit in terms of abnormal estimates of precision in the generative model (Adams et al., 2012). In addition, eye movement signs are ubiquitous in neurology (Anderson and MacAskill, 2013). To take this model forward – to address cardinal oculomotor deficits in psychiatry and neurology – we may need to develop a more complete model that, in addition to accounting for visual and proprioceptive data, accounts for vestibular inputs. This is likely to be important in the development of nystagmus due to cerebellar or brainstem damage (Troost, 1989).

## 8 Conclusion

In this paper, we have demonstrated that active inference provides a sufficient and principled account of oculomotor forces that fulfil prior beliefs about eye movements. By using a generative model that is common to both eyes, we enforce conjugate eye movements. When we map the ensuing Bayesian filtering equations to their associated process theory; namely, predictive coding, we find a connectivity structure that is remarkably consistent with the neuroanatomy of the oculomotor brainstem. Once this anatomical assignment is made, it is possible to simulate saccade-related responses we would expect to record from these regions with an electrode. These were formally very similar to recordings from the homologous anatomical regions in the electrophysiological literature. Finally, we showed that anatomically motivated computational lesions reproduced the eye movement deficits seen in neurological patients.

## Appendix Generalised (Bayesian) filtering

To derive the filtering equations (Friston et al., 2010) in Fig. 3, we first must specify the form of the approximate posterior distribution, $q(\tilde{x},\tilde{v})$. We start by making a mean field approximation: this assumes the full distribution can be obtained through a product of marginal distributions for each temporal derivative ($x^{\left[i\right]}$ means $i^{th}$ derivative of x) of the hidden states and causes.

$$q(\tilde{x},\tilde{v})=\prod_{i}q(x^{\left[i\right]})q(v^{\left[i\right]})$$

If we take one of these marginal posterior distributions, we can relate this to the joint density given by the generative model through Bayes rule. We can then expand this, using a Taylor series, to find an appropriate form for the distribution.

$$\begin{array}{ccc} q(x^{\left[i\right]}) & \approx & p(x^{\left[i\right]}|{\overset{\sim }{x}}^{\backslash i},\overset{\sim }{v},\overset{\sim }{y})\propto p(x^{\left[i\right]},{\overset{\sim }{x}}^{\backslash i},\overset{\sim }{v},\overset{\sim }{y})\\ & \approx & \frac{1}{Z}\exp\left(\ln\;p(\mu_{x}^{\left[i\right]},{\overset{\sim }{x}}^{\backslash i},\overset{\sim }{v},\overset{\sim }{y})+\frac{1}{2}{\left(\mu_{x}^{\left[i\right]}-x^{\left[i\right]}\right)}^{2}P_{x}^{\left[i\right]}+...\right)=N(\mu_{x}^{\left[i\right]},P_{x}^{\left[i\right]})\\ P_{x}^{\left[i\right]} & = & -\partial_{x^{\left[i\right]}x^{\left[i\right]}}\;\ln\;p(\mu_{x}^{\left[i\right]},{\overset{\sim }{x}}^{\backslash i},\overset{\sim }{v},\overset{\sim }{y}) \end{array}$$

The Taylor series expansion reveals the approximate equality between a marginal posterior and a Gaussian distribution (this is also true for $q(v^{\left[i\right]})$). This is known as the Laplace approximation (Friston et al., 2007). Notably, the precision of this distribution is an analytic function of the mean. This means that we only need optimise the mean explicitly.

To find the free energy gradients, we can take the variational derivative with respect to each marginal, and set this to zero. Omitting terms constant terms, this gives:

$$\begin{array}{ccc} \delta_{q(x^{\left[i\right]})}F & = & \;\ln\;q(x^{\left[i\right]})-E_{q(\overset{\sim }{v})q({\overset{\sim }{x}}^{\backslash i})}[\;\ln\;p(\overset{\sim }{y},\overset{\sim }{x},\overset{\sim }{v})]\\ \delta_{q(x^{\left[i\right]})}F & = & 0\Leftrightarrow q(x^{\left[i\right]})=\frac{1}{Z}\exp\left(E_{q(\overset{\sim }{v})q({\overset{\sim }{x}}^{\backslash i})}[\;\ln\;p(y^{\left[i\right]}|x^{\left[i\right]},v^{\left[i\right]})+\;\ln\;p(x^{\left[i+1\right]}|x^{\left[i\right]},v^{\left[i\right]})+\;\ln\;p(x^{\left[i\right]}|x^{\left[i-1\right]},v^{\left[i-1\right]})]\right)\\ \delta_{q(v^{\left[i\right]})}F & = & \;\ln\;q(v^{\left[i\right]})-E_{q({\overset{\sim }{v}}^{\backslash i})q(\overset{\sim }{x})}\;[\;\ln\;p(\overset{\sim }{y},\overset{\sim }{x},\overset{\sim }{v})]\\ \delta_{q(v^{\left[i\right]})}F & = & 0\Leftrightarrow q(v^{\left[i\right]})=\frac{1}{Z}\exp\left(E_{q({\overset{\sim }{v}}^{\backslash i})q(\overset{\sim }{x})}[\;\ln\;p(y^{\left[i\right]}|x^{\left[i\right]},v^{\left[i\right]})+\;\ln\;p(x^{\left[i+1\right]}|x^{\left[i\right]},v^{\left[i\right]})+\;\ln\;p(v^{\left[i\right]})]\right)\\ q({\overset{\sim }{x}}^{\backslash i}) & ≜ & \underset{j\neq i}{\prod }q(x^{\left[j\right]}) \end{array}$$

As the expectations above are with respect to Gaussian distributions, it follows that the optimal means of these distributions are the values that maximise the terms over which the expectation is taken. This allows us to substitute the means into the arguments of the expectations above, and to perform a generalised gradient ascent of the quantity within the expectation.

$$\begin{array}{ccc} \mu_{x}^{\left[i\right]}-\mu_{x}^{\left[i+1\right]} & = & \partial_{x^{\left[i\right]}}\left(\ln\;p(y^{\left[i\right]}|\mu_{x}^{\left[i\right]},\mu_{v}^{\left[i\right]})+\;\ln\;p(\mu_{x}^{\left[i+1\right]}|\mu_{x}^{\left[i\right]},\mu_{v}^{\left[i\right]})+\;\ln\;p(\mu_{x}^{\left[i\right]}|\mu_{x}^{\left[i-1\right]},\mu_{v}^{\left[i-1\right]})\right)\\ & = & -\frac{1}{2}\partial_{x^{\left[i\right]}}\left(\varepsilon_{y}^{\left[i\right]}\Pi_{y}^{\left[i\right]}\varepsilon_{y}^{\left[i\right]}+\varepsilon_{x}^{\left[i\right]}\Pi_{x}^{\left[i\right]}\varepsilon_{x}^{\left[i\right]}+\varepsilon_{x}^{\left[i+1\right]}\Pi_{x}^{\left[i+1\right]}\varepsilon_{x}^{\left[i+1\right]}\right)\\ & = & \partial_{x^{\left[i\right]}}g^{\left[i\right]}\Pi_{y}^{\left[i\right]}\varepsilon_{y}^{\left[i\right]}+\partial_{x^{\left[i\right]}}f^{\left[i\right]}\Pi_{x}^{\left[i\right]}\varepsilon_{x}^{\left[i\right]}-\partial_{x^{\left[i\right]}}\Pi_{x}^{\left[i-1\right]}\varepsilon_{x}^{\left[i-1\right]} \end{array}$$

The second line comes from expanding the log Gaussians, where $\varepsilon_{y}^{\left[i\right]}=y^{\left[i\right]}-g^{\left[i\right]}$, $\varepsilon_{x}^{\left[i\right]}=\mu_{x}^{\left[i+1\right]}-f^{\left[i\right]}$, and $\varepsilon_{v}^{\left[i\right]}=\mu_{v}^{\left[i\right]}-\eta^{\left[i\right]}$. Similarly,

$${\dot{\mu }}_{v}^{\left[i\right]}-\mu_{v}^{\left[i+1\right]}=\partial_{v^{\left[i\right]}}g^{\left[i\right]}\Pi_{y}^{\left[i\right]}\varepsilon_{y}^{\left[i\right]}+\partial_{v^{\left[i\right]}}f^{\left[i\right]}\Pi_{x}^{\left[i\right]}\varepsilon_{x}^{\left[i\right]}-\Pi_{v}^{\left[i\right]}\varepsilon_{v}^{\left[i\right]}$$

Expressing these equalities in terms of generalised coordinates of motion gives the generalised filtering equations shown in Fig. 3.
